# Effects of Ozone Treatment on Personal Protective Equipment Contaminated with SARS-CoV-2

**DOI:** 10.3390/antiox9121222

**Published:** 2020-12-03

**Authors:** Bernardino Clavo, Elizabeth Córdoba-Lanús, Francisco Rodríguez-Esparragón, Sara E. Cazorla-Rivero, Omar García-Pérez, José E. Piñero, Jesús Villar, Angeles Blanco, Cristina Torres-Ascensión, José L. Martín-Barrasa, Jesús M. González-Martin, Pedro Serrano-Aguilar, Jacob Lorenzo-Morales

**Affiliations:** 1 Research Unit, Hospital Universitario Dr. Negrín, 35019 Las Palmas de Gran Canaria, Spain; frodesp@gobiernodecanarias.org (F.R.-E.); jesus.villar54@gmail.com (J.V.); cristina_torres559@hotmail.com (C.T.-A.); jlmbarrasa@gmail.com (J.L.M.-B.); josu.estadistica@gmail.com (J.M.G.-M.); 2 Chronic Pain Unit, Hospital Universitario Dr. Negrín, 35019 Las Palmas de Gran Canaria, Spain; 3 Radiation Oncology Department, Hospital Universitario Dr. Negrín, 35019 Las Palmas de Gran Canaria, Spain; 4 RETIC de Investigación en Servicios de Salud en Enfermedades Crónicas (REDISSEC), Instituto de Salud Carlos III, 28029 Madrid, Spain; pseragu@gobiernodecanarias.org; 5 Instituto Universitario de Investigaciones Biomédicas y Sanitarias (IUIBS), BioPharm Group, Universidad de Las Palmas de Gran Canaria, 35016 Las Palmas de Gran Canaria, Spain; 6 Instituto Universitario de Enfermedades Tropicales y Salud Pública de Canarias, Universidad de La Laguna, La Laguna, 38200 Tenerife, Spain; acordoba@ull.edu.es (E.C.-L.); sara_ingenio@hotmail.com (S.E.C.-R.); omargp6@gmail.com (O.G.-P.); jpinero@ull.edu.es (J.E.P.); jmlorenz@ull.edu.es (J.L.-M.); 7 Departamento de Medicina Interna, Dermatología y Psiquiatría, Universidad de La Laguna, La Laguna, 38200 Tenerife, Spain; 8 Red Cooperativa de Enfermedades Tropicales (RICET), Instituto de Salud Carlos III, 28029 Madrid, Spain; 9 Departamento de Obstetricia, Ginecología, Pediatría, Medicina Preventiva y Salud Pública, Toxicología, Medicina Legal y Forense y Parasitología, Universidad de La Laguna, La Laguna, 38200 Tenerife, Spain; 10 CIBER de Enfermedades Respiratorias, Instituto de Salud Carlos III, 28029 Madrid, Spain; 11 Chemical Engineering & Materials Department, Universidad Complutense, 28040 Madrid, Spain; ablanco@ucm.es; 12 Animal Infectious Diseases and Ictiopathology, Universitary Institute of Animal Health and Food Safety (IUSA), Universidad de Las Palmas de Gran Canaria, 35413 Arucas, Spain; 13 Servicio de Evaluación y Planificación del Servicio Canario de Salud (SESCS), 38109 Santa Cruz de Tenerife, Spain; 14 Red de Agencias de Evaluación de Tecnologías Sanitarias y Prestaciones del Sistema Nacional de Salud (RedETS), 28071 Madrid, Spain

**Keywords:** COVID-19, decontamination, personal protective equipment, oxidative stress, ozone treatment, reactive oxygen species, SARS-CoV-2

## Abstract

Background: Severe acute respiratory syndrome coronavirus 2 (SARS-CoV-2) is causing profound health, economic, and social problems worldwide. Management of personal protective equipment (PPE) and its potential limited availability have created concerns about the increased risks for healthcare professionals at hospitals and nursing homes. Ozone is a powerful oxidant agent. The objectives of this study were to examine the effects of ozone treatment on PPE contaminated with SARS-CoV-2, and to explore whether relative humidity could modify those effects. Methods: PPE contaminated by heat-inactivated SARS-CoV-2 were treated with different ozone concentrations, exposure times, and relative humidity conditions. SARS-CoV-2 gene amplification was assessed by real-time polymerase chain reaction. Results: There was no amplification of SARS-CoV-2 in PPE after the following ozone exposures: 30 s at 10,000 ppm (20 g/m^3^), 5 min at 4000 ppm, and 10 min at 2000 ppm. At lower ozone concentrations, 4–12 ppm (0.008–0.024 g/m^3^), the effects were highly dependent on the relative humidity conditions. Conclusions: Oxidative stress induced by ozone exposure eliminated heat-inactivated SARS-CoV-2 in different PPE components under appropriate exposure times, ozone concentrations, and relative humidity conditions. These findings could have implications in decreasing the risk of contamination associated with personal protective equipment management and in increasing its availability. Further research in the original SARS-CoV-2 strain is guaranteed.

## 1. Introduction

Coronavirus disease 2019 (COVID-19) is caused by severe acute respiratory syndrome coronavirus 2 (SARS-CoV-2) and has rapidly evolved into a worldwide pandemic affecting millions of people and causing more than one million deaths at the time of writing this manuscript, challenging the response capacity of the healthcare system. Furthermore, transmission to healthcare professionals and caregivers is an additional threat in hospitals and nursing homes [1,2,3].

The World Health Organization (WHO) declared COVID-19 as a pandemic on 11 March 2020 [4]. Initially, the most affected countries were China, Italy, and Spain, with large and fast dissemination associated with a sudden high requirement of personal protective equipment (PPE), low availability of mechanical ventilators and intensive care unit beds, and an increased risk of infection among patients and healthcare professionals [1,2,3]. The global impact of this health problem led to a “Call for Ideas for Conserving Supply of PPE” by the editors of the Journal of the American Medical Association (JAMA) on 20 March 2020 [5]. We answered this call with a Comment published three days later (Comment #224) [5] describing the potential use of ozone for PPE decontamination.

Ozone is the third most potent oxidant agent after fluor and persulfate and can induce oxidative stress in living organisms. For a century, this property has been used for water treatment, and ozone is considered by the WHO as one of the best biocides against microorganisms [6]. Additionally, it has been previously reported that ozone can kill viruses such as Enterovirus [7], Poliovirus, Rhinovirus, and Murine coronavirus [8,9], all of them from the same Group IV as SARS-CoV-2 (according to the Baltimore classification), which is characterized by a positive-sense single-stranded RNA.

This report shows for the first time a controlled study on the effects of ozone exposure on several PPE surfaces contaminated with SARS-CoV-2.

## 2. Materials and Methods

This study was approved by the Regional Ethics Committee (Comité Ético de Investigación Clínica con Medicamentos, Hospital Universitario de Gran Canaria Dr. Negrín, Las Palmas, Spain, code #2020-183-1 COVID-19).

In this study, we evaluated the viricidal effects of several approaches of ozone treatment of PPE gowns and FFP2 face masks (filtering facepiece with minimum efficiency of > 92%) contaminated with SARS-CoV-2, including changes in exposure times and ozone concentrations. The secondary objective was to explore whether relative humidity could modify the effects of ozone on SARS-CoV-2. We hypothesized that SARS-CoV-2 present on PPE could be destroyed by ozone under appropriate ozone concentrations, exposure times, and relative humidity.

### 2.1. Study Design, Samples, and Outcome Assessment

This prospective study was conducted in two phases. In the first phase, we evaluated a range of short exposure times from 30 s to 10 min at high ozone concentrations, from 500 to 40,000 parts of ozone per million parts of air (ppm), equal to 1–80 g/m^3^. These high ozone concentrations are only possible in small volumes. In the second phase, we examined the impact of changes in relative humidity on the effects of low ozone concentrations, which have been used for decontamination of large rooms and warehouses for several years.

PPE gowns and face masks were contaminated with SARS-CoV-2 strain 2019-nCoV/USA-WA1/2020, inactivated by heating to 65 °C for 30 min (ATCC^®^ VR-1986HK™). Samples around 20 × 10 mm were contaminated with a drop of 10 µL of SARS-CoV-2 at a concentration of 1000 copies/µL. At each exposure time and ozone concentration, two samples of each virus-infected material (gowns or face masks) were analyzed in the first phase and only one sample of each infected material in the second phase of the study. For each duplicated infected sample that was treated with ozone at each point concentration, we obtained one swab, which was maintained in medium UTM 3ML RT (COPAN, Biomerieux) for transportation. Then, from each swab, two genomic analyses by real-time polymerase chain reaction (RT-PCR) were performed for each swab. The primary outcome measure was to evaluate the disappearance of SARS-CoV-2 gene amplification assessed by RT-PCR.

### 2.2. Ozone Exposure Details

Ozone exposure of PPE components was performed at the Hospital Universitario Dr. Negrin (Las Palmas de Gran Canaria, Spain). For the first phase, ozone was produced by medical ozone generators (Ozonobaric P^®^, Sedecal, Madrid, Spain). These devices generate ozone from medical-grade oxygen, obtaining an O_3_/O_2_ gas mixture between 500 and 40,000 ppm (1–80 g/m^3^). Room temperature (22.0–23.7 °C) and relative humidity (53–65%) were controlled by the air-conditioning system of the hospital. In this phase, samples contaminated with SARS-CoV-2 were introduced with a 60 mL syringe; then, the air was expelled, and ozone was introduced at different concentrations and exposure times.

For the second phase, we used a portable and specifically designed ozonation chamber (UVOZ^®^, Lighting Dynamic Technology, Las Palmas de Gran Canaria, Spain) of 200 × 100 × 100 cm^3^ with an industrial ozone generator, where ozone is obtained from ambient air, and a humidifier. Here, we analyzed lower ozone concentrations for longer exposure times (from 30 to 50 min). The ozone concentration measured in the ozonation chamber was 8–12 ppm (0.016–0.024 g/m^3^) under standard relative humidity (62–63%), and 4–6.5 ppm (0.008–0.013 g/m^3^) under forced 99% relative humidity conditions. The ozonation chamber kept the concentration alternating on/off periods of the ozone generator, to compensate for the spontaneous decomposition of ozone to oxygen (half-life of 40 min at 20 °C and 25 min at 30 °C) [10].

### 2.3. Real-Time Polymerase Chain Reaction (RT-PCR)

RT-PCR was used to quantify viral RNA by the Instituto Universitario de Enfermedades Tropicales y Salud Pública de Canarias (La Laguna, Tenerife, Spain), according to Spanish guidelines for biosafety level-2 facilities. Once processed, all swabs and tubes were treated with VirkonTM (Biotein SL, Gran Canaria, Spain) for a minimum of 2 h and autoclaved. Viral RNA was extracted using the Maxwell 16S Viral RNA Mini Kit (Promega, Madrid, Spain) following the manufacturer’s recommendation. Resulting RNA was eluted in 50 µL of elution buffer until further use for RT-PCR. The TaqPath™ COVID-19 CE-IVD RT-PCR Kit (Applied Biosystems, Thermo Fisher Scientific, Madrid, Spain) was used to perform the RT-PCR assays following the manufacturer’s instructions, in combination with the TaqPathTM 1-Step RT-qPCR Master Mix. This kit contains a set of TaqPath™ COVID-19 Assay Multiplex for the qualitative detection and characterization of SARS-CoV-2 RNA. Briefly, the kit included three assays that target SARS-CoV-2 genes (Gene ORF1ab, N Protein, S Protein), and as a control of RNA extraction, the MS2 Phage Control. It also contains a positive TaqPath™ COVID-19 Control. All the experiments were performed in duplicate in a QuantStudio3™ Real-Time PCR System (Applied Biosystems).

## 3. Results

There was no SARS-CoV-2 gene amplification neither in PPE gowns nor face masks at ozone concentrations higher than 10,000 ppm (20 g/m^3^) after 30 s.

**Figure 1:**
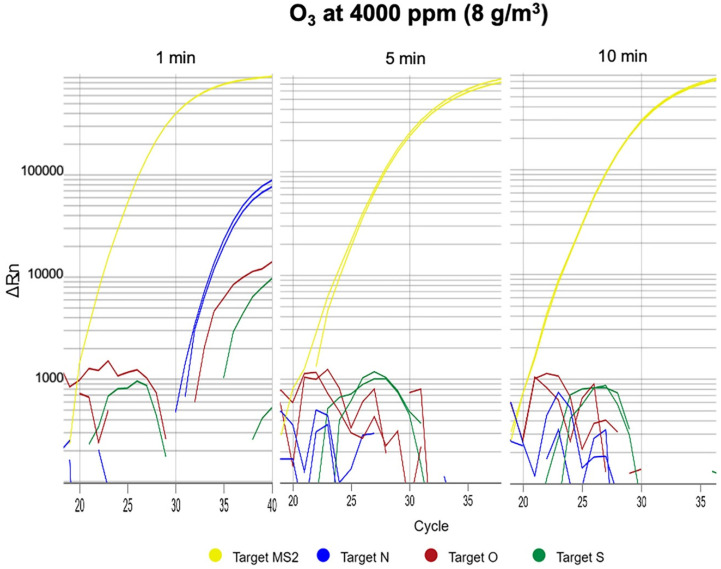
Personal protective equipment (PPE) gown samples after ozone exposure at 4000 ppm (8 g/m^3^) for 1, 5, and 10 min. After 1 min, we could still observe amplification of 1 of the 3 genes in PPE gown samples. There was no gene amplification with ozone exposure for 5 or 10 min.

We observed virus amplification in PPE gowns but not in face masks at 4000 ppm (8 g/m^3^) after 30 s of ozone exposure. After increasing ozone exposure to 1 min, we still observed amplification of one of the three genes in PPE gown samples, but not in face masks. However, after 5 min, there was no SARS-CoV-2 gene amplification in either PPE gowns or face masks (Figure 1).

**Figure 2:**
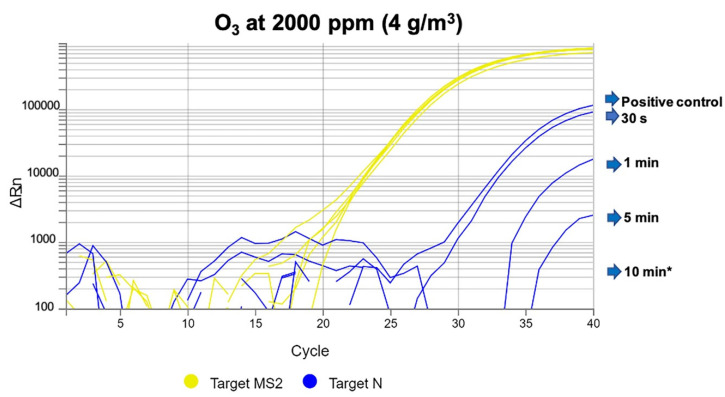
Severe acute respiratory syndrome coronavirus 2 (SARS-CoV-2) (N gene amplification) detected in PPE gown samples after ozone exposure at 2000 ppm (4 g/m^3^) after 30 s, and 1, 5, and 10 min. The image shows a relationship between increase in exposure times and progressive decrease in gene amplification, with increasing threshold cycle values. * No gene amplification was detected at 10 min of exposure.

After 30 s or 1 min under ozone exposure at 2000 ppm (4 g/m^3^), SARS-CoV-2 was detected in PPE gown and face mask samples, although gene amplification was lower in the face mask samples by increasing threshold cycle values. After 5 min, there was still amplification of one gene in PPE gown samples, but there was no virus detection in face mask samples. At 10 min of ozone exposure, there was no SARS-CoV-2 gene amplification in either PPE gowns or face masks (Figure 2).

**Figure 3:**
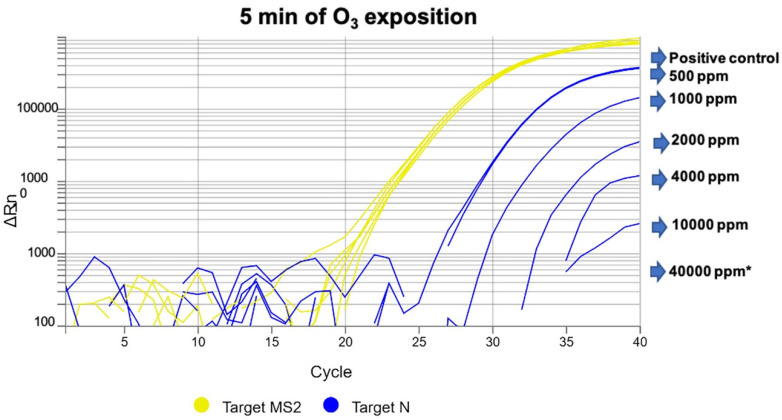
SARS-CoV-2 (N gene amplification) detected in PPE gown samples after 5 min of ozone exposure at different ozone concentrations. The image shows a relationship between the increase in ozone concentration and progressive decrease in gene amplification, with increasing threshold cycle values. * No gene amplification was detected at 40,000 ppm (80 g/m^3^).

Ozone exposure at 1000 ppm (2 g/m^3^) and 500 ppm (1 g/m^3^) for 5 or 10 min resulted in viral gene amplification in PPE gowns and face masks. Figure 3 shows gene amplification detected after 5 min of exposure at different ozone concentrations.

Table 1 summarizes the results with different ozone concentrations at different exposure times in the first phase of the study.

**Figure 4:**
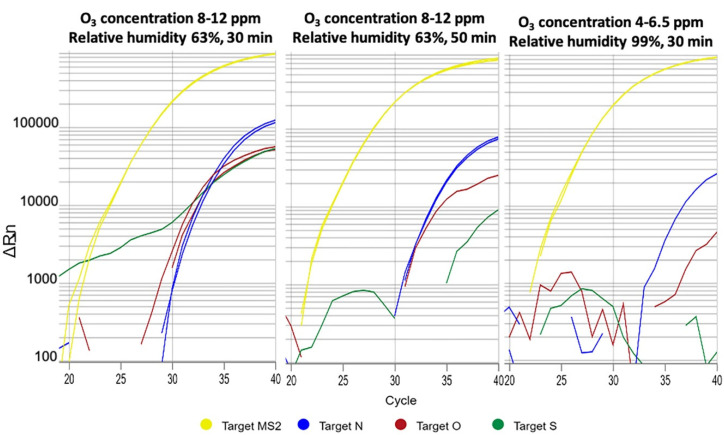
Gene amplification detected in PPE gown samples under 63% relative humidity (our standard condition) and under forced 99% relative humidity (in the ozonation chamber). Note that under the same conditions of relative humidity and ozone concentration, the effect was higher at 50 min (Middle) than at 30 min (Left), although both showed virus detection. (Right) Despite the lower ozone concentration (4–6.5 ppm, 0.008–0.013 g/m^3^), under 99% relative humidity, there was no gene amplification after 30 min, suggesting that at higher relative humidity, ozone has a better viricidal effect against PPE gowns contaminated with SARS-CoV-2.

After 30 min in the ozonation chamber with exposure at 8–12 ppm (0.016–0.024 g/m^3^) under 63% relative humidity, virus gene amplification was detected in PPE gowns and face masks. When the time was increased to 50 min, the virus was still present, although a decrease in all gene amplifications was observed. In the ozonation chamber with exposure at 4–6.5 ppm (0.008–0.013 g/m^3^) under 99% relative humidity, SARS-CoV-2 was completely eliminated from PPE gown samples after 30 and 50 min. However, gene amplification was present in face masks after 30 and 50 min (Figure 4).

## 4. Discussion

This report shows for the first time that: (1) oxidative stress induced by appropriate combinations of concentration and time exposure of ozone can eliminate SARS-CoV-2 in PPE gowns and face masks, (2) SARS-CoV-2 disinfection dosages vary for the different studied materials, and (3) the viricidal effect of ozone on SARS-CoV-2 depends on relative humidity. Therefore, optimal dosages need to be optimized depending on the required disinfection level, the treated material, and the local relative humidity.

Previous studies have reported that SARS-CoV-2 survival depends on the type of surface under examination. For instance, the virus was not able to survive for more than 4 h on copper or 24 h on cardboard at 21–23 °C and 40% relative humidity. In contrast, in less porous materials such as glass, steel, and plastic, the virus persists for up to 72 h [11]. Longer survival (more than 7 days) has been reported on the outer surface of surgical masks [12]. This, along with the increased requirements and low availability of PPE during the COVID-19 pandemic, explains the increased risk for exposed healthcare professionals [1,2,3]. While most PPE components (gowns, masks, gloves) were designated for single use, during the COVID-19 pandemic, reutilization was considered, requiring effective and fast treatment methods conserving material properties. This could reduce consumption (increasing availability) and reduce waste products (decreasing environmental impact).

In the first phase of our study, we evaluated high/moderate ozone concentrations, which are only available at small volumes. We observed that ozone concentrations of 4000–10,000 ppm (8–20 g/m^3^) have a viricidal effect after short-time exposure (from half to few minutes) on PPE gowns and face masks contaminated with SARS-CoV-2. These results also point to the possibility of PPE decontamination without undressing, as we have previously suggested (Comment #224) [5], and offer two potential applications to evaluate: (1) to avoid or decrease PPE undressing and using a new PPE when healthcare professionals move from one risk patient or risk area to another, saving PPE while decreasing the number of PPE-undressing procedures, and (2) to treat PPE before the undressing procedure. Both of these potential applications could decrease the risk associated with the PPE-undressing procedure for healthcare professionals. However, this should be done while avoiding ozone breathing, to prevent lung damage induced by the oxidative stress. Further research is required for validation and establishing appropriate implementation.

In the second phase of our study, we examined whether relative humidity could modify the effects of low ozone concentrations at higher exposure times, as usually used to decrease contamination by other pathogens in large volumes (rooms and warehouses). Some findings were unexpected. First, under 63% relative humidity, the virus was expected to be present after 30 min, but not after 50 min. A press release (14 May 2020) from Nara Medical University (Japan) described 99.90% to 99.99% of SARS-CoV-2 inactivation after 55 min at 6 ppm (0.012 g/m^3^) [13]. However, that unpublished study evaluated virus inactivation after ozone exposure using virus cultures, while we examined SARS-CoV-2 gene amplification and did not evaluate inactivation. Second, under 99% relative humidity, better results were obtained, as expected, in PPE gowns. However, results in face masks did not improve, which was unexpected. A plausible explanation is that at 99% relative humidity, there are high condensation levels in all surfaces inside the ozonation chamber and on PPE surfaces. Condensation in masks likely hindered the contact between ozone and virus. Third, although the ozonation-chamber program was the same with standard or high relative humidity, it measured near half ozone concentration under 99% relative humidity (8–12 ppm (0.016–0.024 g/m^3^) vs. 4–6.5 ppm (0.008–0.013 g/m^3^), respectively). This could also be related to condensation inside the ozonation chamber, since ozone is 10 times more soluble in water than oxygen [10]. In large ozonation chambers with other viruses, it has been reported that ozone has higher viricidal effects at higher relative humidity [8]. This is in agreement with a higher coronavirus inactivation rate on surfaces with increased relative humidity [14]. This is a relevant issue because the ozone effects could differ in different cities or hospitals under different environmental conditions, and if required, these conditions could be modified (e.g., by increasing the relative humidity in ozonation chambers) to obtain better or faster results. Our findings in the second phase were obtained using an ozonation chamber of 200 × 100 × 100 cm^3^ with a moderated volume (2000 L), where five complete PPE gowns could be introduced, simultaneously maintaining ozone exposure for all gown surfaces for SARS-CoV-2 decontamination. It is necessary to always maintain appropriate ozone concentrations and exposure times depending on relative humidity. Larger volumes (e.g., a room) can facilitate the treatment of a higher number of full PPE gowns, but at the expense of a potential decrease in ozone concentration, which should be compensated by increasing ozone generation and/or increasing exposure time. This is another area requiring further research.

The mechanisms of action of ozone depend on its strong oxidant effect (2.07 V oxidation vs. 1.36 for chlorine), which explains its induced oxidative stress and its broad antimicrobial spectrum. This effect is mediated by peroxidation and damage of polyunsaturated fatty acids from cell membranes (in bacteria, mold, yeast, and spores), proteins (especially S-proteins), and lipids of capsid and viral envelopment, as well as by inducing indirect damage to DNA and RNA [15]. Damage to RNA is the major mechanism of Poliovirus-1 and Echovirus inactivation [16,17]. Further details about the oxidant effects of ozone in SARS-CoV-2 have been recently reported [18]. The WHO considers ozone to be one of the best biocides against microorganisms [6]. In comparison with liquid disinfection systems, ozone has some advantages that could be exploited in some circumstances: (1) as a gas, ozone has easier access to small or irregular surfaces, (2) when its effect has finished, there are no residual subproducts that can induce irritation, and (3) it could be potentially used in some materials or surfaces that are not moisture-tolerant [19].

We acknowledge some limitations to our study. First, we are not sure whether the behavior of the heated-inactivated SARS-CoV-2 against ozone could be different from the actual SARS-CoV-2 and further research in the original SARS-CoV-2 strain is needed. Our findings only show concentration and times for complete elimination of virus genes, while inactivation could also be useful to avoid or decrease the transmission of this virus. Since this study was based on a commercial heated-inactivated SARS-CoV-2, we could not evaluate the required concentration and time for virus inactivation, which should be lower than that required for virus elimination. In our study, SARS-CoV-2 inactivation probably corresponds to those lower exposure times and ozone concentrations showing (1) non-amplification for one or two genes, or (2) virus presence, but requiring higher thermal cycles of gene amplification or lower survival logarithms. This would be in agreement with times and ozone concentrations previously published about the viricidal effect of ozone in other viruses. Second, PPE components were contaminated by a droplet with high virus concentration (1000 viruses/µL) and not by spraying. Thus, the virus concentration in the samples and swabs was probably much higher than the concentration present on PPE surfaces under standard conditions, and the effects of ozone in our study could have been underestimated [20]. Third, for the potential decontamination with ozone of many PPE gowns at the same time, it would require large volumes and consequently low ozone concentrations and larger exposure times than those found in the first phase of our study. However, in the second phase, ozone exposure at 4–6 ppm (0.008–0.013 g/m^3^) for 30 min under high relative humidity in the ozonation chamber was enough to eliminate SARS-CoV-2. Fourth, we did not analyze the effects of ozone on aerosols containing SARS-CoV-2. However, the viability of SARS-CoV-2 in aerosols is significantly shorter (around three hours) than on plastic surfaces [11]. Virus suspension in the air is only possible in microdroplets with a lower virus load than the drop we used in our study. Then, lower combinations of concentration and time exposure of ozone than those described in our study could potentially be useful for air decontamination. This hypothesis agrees with studies showing that confirmed COVID-19 cases were lower when ambient ozone concentrations were higher [21]. This potential application of ozone could decrease the risk of aerosol and fomite transmission in large rooms with many people or high-risk areas (such as hospitals or nursing homes). However, this potential use of ozone has not been demonstrated yet, and further studies to confirm this effect are needed. Fifth, the extremely high humidity (99%) used in the second phase of our study is not usual or workable, and it led to condensation on the internal surfaces of the ozonation chamber. It was chosen to better explore whether relative humidity levels could modify ozone effects, which was observed in gowns. The effect of slightly lower levels (80–90% relative humidity) could be explored to decrease exposure times in ozone chambers, at least in selected materials when rooms or cities have low relative humidity.

Finally, we want to mention that (i) the effect of ozone in very dirty materials with thicker spots or biofilm contents could be reduced because of lower penetration or inactivation by biological antioxidants, and (ii) breathing air with ozone concentration above the recommended upper limit of 0.08–0.10 ppm (0.16–0.20 mg/m^3^) must be avoided [22,23].

## 5. Conclusions

In summary, this study shows, for the first time, that ozone can successfully eliminate SARS-CoV-2 from the surface of PPE gowns and face masks, with a viricidal effect depending on ozone concentrations, exposure times, and relative humidity. Our findings agree with previous studies on the viricidal effect of ozone in other viruses and augur well for its potential use in the management and/or reutilization of PPE components to prevent or decrease the risk of contamination by SARS-CoV-2 in hospitals, nursing homes, and other environments. Additional studies are needed for implementing the use of ozone in pandemics.

## 6. Patents

The ozonation chamber UVOZ^®^ used in the second phase has been patented (No. U202030703) by Lighting Dynamic Technology, S.L., with the participation of 10 of the authors of this manuscript: B.C., E.C.-L., F.R.-E., J.E.P., J.V., A.B., J.L.M.-B., J.M.G.-M., P.S.-A., and J.L.-M.

## Figures and Tables

**Table 1:** SARS-COV-2 detection in PPE gown and face-mask samples analyzed at different ozone concentrations and exposure times in the first phase of the study. X: SARS-CoV-2 negative gene amplification by real-time polymerase chain reaction (RT-PCR), √: SARS-CoV-2 detected by RT-PCR (amplification of 3 virus-specific genes), √*: SARS-CoV-2 detected by RT-PCR (amplification of 1 or 2 virus genes), n.a.: not applicable.

Materials	PPE Gowns				Face Masks			
Time	30 s	1 min	5 min	10 min	30 s	1 min	5 min	10 min
Ozone Concentration								
40,000 ppm (80 g/m^3^)	X	X	n.a.	n.a.	X	X	n.a.	n.a.
10,000 ppm (20 g/m^3^)	X	X	n.a.	n.a.	X	X	n.a.	n.a.
4000 ppm (8 g/m^3^)	√	√*	X	X	X	X	X	X
2000 ppm (4 g/m^3^)	√	√	√*	X	√	√*	X	X
1000 ppm (2 g/m^3^)	√	√	√	√	√	√	√	√
500 ppm (1 g/m^3^)	√	√	√	√	√	√	√	√
